# New Approach in the Determination of a Suitable Directionally Coarsened Microstructure for the Fabrication of Nanoporous Superalloy Membranes Based on CMSX-4

**DOI:** 10.3390/ma16103715

**Published:** 2023-05-13

**Authors:** Janik Marius Lück, Joachim Rösler

**Affiliations:** Institute for Materials Science, Technische Universität Braunschweig, Langer Kamp 8, 38106 Braunschweig, Germany

**Keywords:** CMSX-4, superalloy membranes, creep test, directional coarsening, premix membrane emulsification

## Abstract

The pore size of nanoporous superalloy membranes produced by directional coarsening is directly related to the γ-channel width after creep deformation, since the γ-phase is removed subsequently by selective phase extraction. The continuous network of the γ′-phase thus remaining is based on complete crosslinking of the γ′-phase in the directionally coarsened state forming the subsequent membrane. In order to be able to achieve the smallest possible droplet size in the later application in premix membrane emulsification, a central aspect of this investigation is to minimize the γ-channel width. For this purpose, we use the 3*w*_0_-criterion as a starting point and gradually increase the creep duration at constant stress and temperature. Stepped specimens with three different stress levels are used as creep specimens. Subsequently, the relevant characteristic values of the directionally coarsened microstructure are determined and evaluated using the line intersection method. We show that the approximation of an optimal creep duration via the ${3w}_{0}$-criterion is reasonable and that coarsening occurs at different rates in dendritic and interdendritic regions. The use of staged creep specimens shows significant material and time savings in determining the optimal microstructure. Optimization of the creep parameters results in a γ-channel width of 119 ± 43 nm in dendritic and 150 ± 66 nm in interdendritic regions while maintaining complete crosslinking. Furthermore, our investigations show that unfavorable stress and temperature combinations favor undirectional coarsening before the rafting process is completed.

## 1. Introduction

Previous studies by Kohnke et al. [1] have shown that there is a promising application opportunity for nanoporous superalloy membranes in the field of premix membrane emulsification, a process that can be used to produce nanoemulsions, e.g., for pharmaceutical applications. Particularly promising results were achieved for directionally coarsened γ′-membranes based on the single crystalline superalloy CMSX-4. Key processing steps to produce this kind of superalloy membrane are (i) solution and precipitation heat treatment of CMSX-4 resulting in cubic γ′-particles embedded in the γ-matrix; (ii) directional coarsening, also referred to as rafting, of the γ′-particles by creep deformation to a bicontinuous γ/γ′-microstructure; and (iii) selective phase extraction of the γ-phase so that a nanoporous material consisting of the γ′-phase and channel-like porosity at the location of the dissolved γ-phase results. In premix membrane emulsification, a coarse emulsion is then pushed through the membrane, resulting in droplet size reduction. To achieve the finest possible droplet size and a narrow droplet size distribution during this process, it is of great importance to reduce the γ-channel width, i.e., the subsequent pore size, by adjusting the process parameters in the creep test [1,2,3,4,5]. Therefore, for the production of nanoporous superalloy membranes with a directionally coarsened microstructure, the process of rafting is of central importance. This depends on several factors such as the direction of loading, the γ′-volume fraction, and the misfit, which is defined as $\delta =2\left(a_{\gamma^{\prime }}-a_{\gamma }\right)/(a_{\gamma^{\prime }}+a_{\gamma })$ with a_γ_ and a_γ′_ being the lattice parameters of the γ- and γ′-phase, respectively [6,7,8,9]. Single crystalline Ni-based superalloys such as CMSX-4 have a negative misfit of the magnitude of δ ≈ −0.001 at room temperature. Due to the different thermal expansion coefficients of γ and γ′, the misfit δ becomes increasingly negative with increasing temperature [6,10,11]. If the starting material, which contains cube-shaped γ′-particles (L_12_ crystal structure) coherently embedded in the fcc γ matrix, is loaded in the [001]-direction at elevated temperatures, a change in microstructure occurs and a raft structure is formed [4,12]. This is due to the introduction of dislocations in the γ-channels between the γ′-precipitates. In case of a negative misfit δ, the superposition of applied tensile force and internal misfit stresses leads to shear stresses, which are highest in the horizontal γ-channels so that plastic deformation starts there. Dislocations of type $a/2<0\overset{-}{1}1>\{111\}$ preferentially bulge through the γ-channels leaving interfacial dislocations of screw or mixed character in the γ/γ′-interfaces [13,14,15,16]. The dislocations introduced in this way shift the misfit δ to more positive or negative values in the horizontal and vertical channels, respectively [17]. For alloys with negative misfit, this means that the associated coherency stresses are reduced in the horizontal channels while they are elevated in the vertical ones. Consequently, it becomes energetically favorable to widen the horizontal channels at the expense of the vertical ones, leading eventually to the coalescence of the γ′-precipitates at the vertical γ-channels. This process is called rafting [1,4,6,12,14,15,16]. Thus, a negative misfit δ results in rafts oriented perpendicular to the tensile loading direction, referred to as type-N. If the misfit δ is positive, rafts form parallel to the load direction and are referred to as type-P [7]. Altogether, a bicontinuous γ/γ′-network is formed, where both phases interpenetrate each other while each phase is connected in itself [3]. This understanding led to the invention and development of nanoporous superalloy membranes [3,5].

During past research on the fabrication of nanoporous superalloy membranes based on CMSX-4, mainly directional coarsening and crosslinking at a temperature of 1000 °C and a tensile stress of 170 MPa was investigated. The γ-channel widths obtained were 250–400 nm [18]. An important component of this investigation is the γ-channel widening rate experimentally determined by Epishin et al. [19,20] for the horizontal γ-channels of dendritic regions. Two different rates ${\dot{w}}_{1}$ and ${\dot{w}}_{2}$ were found for the process of raft formation and subsequent coarsening, respectively. Furthermore, it was found that at the time the raft formation is completed, the channel width of the horizontal channels is about three times the initial channel width $w_{0}$ in the precipitation heat-treated condition. This was attributed to the fact that there are twice as many vertical channels as horizontal ones and that the entire γ-phase of the closing vertical channels is transferred to the horizontal ones. This is called the ${3w}_{0}$-criterion [19]. Taking these conditions into account, an optimal heat treatment was therefore worked out in a previous study, combining the most regular arrangement and angularity of the γ′-particles with the minimum size $w_{0}$ of the γ-channels. The optimized precipitation heat treatment at 1140 °C for 30 min results in a γ′-particle size of 224 ± 52 nm and a γ-channel width of 35 ± 19 nm [21].

Using the above-mentioned heat path development and the resulting initial microstructure, we investigate in this work the influence of stress and time on the microstructure development during creep deformation with the aim of identifying optimal creep parameters for the production of superalloy membranes with the smallest possible γ-channel width. Since the subsequent membranes are subjected to mechanical stress in premix membrane emulsification, the selection of the process parameters should include an additional increase in strength via an increased γ′-content. As the γ′-volume fraction depends on the temperature and previous investigations were carried out at 1000 °C, the temperature in the creep tests will be reduced to 950 °C. According to Epishin et al. [22], this increases the γ′-volume content from 70% to 72%. To compensate for the reduced channel widening rate due to the reduced temperature, the stress in the creep tests is increased up to 250 MPa [20]. In order to be able to investigate the influence of different creep stresses at a constant temperature, stepped creep specimens are used. These have three different diameters in three sections so that stresses of 250 MPa, 183 MPa, and 140 MPa can be set in one sample. A similar variant with different dimensions has already been used by Cheng et al. [23] in tensile and compressive creep tests. Epishin et al. [19] used a so-called flat wedge-shaped sample. The designs differ in that we used clearly separated sections with transition radii rather than a rectangular, continuously converging cross-section. However, in this investigation, the combination of 950 °C and 250 MPa is decisive for the subsequent membrane production. In summary, this investigation pursues two goals in the context of superalloy membrane development:

1. To identify suitable creep parameters for the fabrication of nanoporous superalloy membranes with the application target of premix membrane emulsification with minimized pore size.
2. To investigate if stepped creep specimens are suitable for the fast and cost-effective investigation of multiple stress and temperature combinations in membrane development.

## 2. Methodology

### 2.1. Materials and Processing

**Figure 1:**
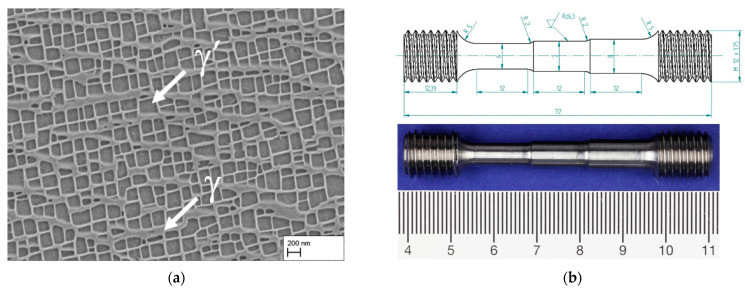
(**a**) γ/γ-microstructure of the CMSX-4 creep samples used after homogenization and precipitation heat treatment. The γ’-particle size is 224 ± 52 nm, the γ-channel width 35 ± 19 nm, (**b**) technical drawing of the stepped creep specimens used in this study (top) and exemplary stepped creep specimen made of CMSX-4 before the creep test (bottom).

To investigate the influence of creep duration and stress on the microstructure evolution during directional coarsening, the single-crystalline Ni-based superalloy CMSX-4 was used. For this purpose, three out of four creep specimens were manufactured from rods with a length of 220 mm and a diameter of 21 mm produced by Access Aachen e.V. using the Bridgeman process (Specimen ID starting with “A-”). The deviation of the [001]-orientation from the rod centerline was determined by Röntgenlabor Eigenmann using the Laue diffraction method. It was less than 10° for the used rods. One of the four samples used was made from a single crystal plate. The deviation of the plate center axis from the [001]-orientation was measured using electron backscatter diffraction (EBSD) in a scanning electron microscope (FEI Helios NanoLab 650) and was 6–10° (Specimen ID starting with “I-”). All samples were heat treated using the following homogenization (HT) and precipitation heat treatment: 1277 °C/2 h + 1288 °C/3 h + 1296 °C/3 h + 1304 °C/2 h + 1313 °C/2 h + 1316 °C/2 h + 1318 °C/2 h + 1321 °C/2 h + AC to RT + 1140 °C/30 min + AC to RT (AC: Air cooling). The precipitation heat treatment at 1140 °C for 30 min is the result of previous studies on γ′-precipitate size and morphology as mentioned before [21]. For completeness, Figure 1a shows the γ/γ′-microstructure in the precipitation-hardened state. The average edge length of the cubic γ′-particles is 224 ± 52 nm, and the channel width between the γ′-particles 35 ± 19 nm. Stepped creep specimens were then fabricated with a total length of 72 mm. The three sections along the gauge length have diameters of 6, 7, and 8 mm, and the specimens have a metric M12 × 1.75 thread at each end. Figure 1b shows the technical drawing of the creep test sample and a manufactured sample from CMSX-4. Table 1 summarizes the test conditions of the investigated stepped creep specimens. The minimal creep duration of 140 h at a stress of 250 MPa was estimated using the channel widening rate ${\dot{w}}_{1}=5.25\times 10^{-1}\frac{nm}{h}$ determined experimentally by Epishin et al. [19,20] in dendritic regions for that test condition. Creep tests were carried out with a constant weight applied to the creep frame. Following the creep test, the samples were cleaned and the three sections were separated. For further investigations of the microstructure, each section was cut in half parallel to the [001] direction.

In order to be able to check the crosslinking in dendritic and interdendritic areas, sheets for the production of membranes were taken from the creep specimens. The sheets were ground to a thickness of approximately 0.3 mm after cutting (including P2500). Electrochemical etching was used to dissolve the γ-phase, leaving the γ′-phase as a solid membrane and a channel-like porosity in place of the γ-phase. A solution of 800 mL H_2_O, 8 g (NH_4_)_2_SO_4_, and 8 g C_6_H_8_O_7_ and an extraction voltage of 1.3 V were used. For a detailed description as well as a detailed experimental setup, see [5]. The specimens with the specimen ID A-CMSX-4/140h/250, A-CMSX-4/170h/250, A-CMSX-4/310h/250, and I-CMSX-4/506h/250 were investigated with this method.

### 2.2. Microstructure Analysis

For the investigation and analysis of the microstructure, the samples were first ground and polished up to and including oxide polishing suspension (OPS). Then the γ′-phase was etched with the help of molybdic acid (1 part H_2_O, 1 part HCl (37%), 1 part HNO_3_, 1 weight-% MoO_3_) for 3 s to obtain sufficient contrast in the scanning electron microscope (SEM). For each condition, five SEM images were taken with a ZEISS LEO 1550 GEMINI at a magnification of 8 k and then used for the measurement with the line intersection method. To be able to carry out the measurement of the γ/γ′-microstructure, first, a binarization was necessary. For this purpose, after cropping the image to 688 × 688 pixels, the software ImageJ [24] and the plugin Trainable Weka Segmentation [25] were used. The plugin is trained by the user’s input; in the broadest sense, this training is conducted by determining which pixels with which grey values belong to the γ-phase or γ′-phase. The software can use various settings, such as taking the neighboring pixels into account, to make the distinction between the adjacent pixels. Since the recorded images were usually taken with identical brightness and contrast settings for each creep state, the software only had to be trained once for each state to the corresponding settings. Afterward, the image was imported into a program for the line intersection method.

In the line intersection method, a defined set of parallel lines is placed in the binarized image in an angular range of 0° to 180° degrees. The software then detects where the line is interrupted by black or white areas and measures the length of these areas according to the previously defined distance between adjacent pixels. This method was carried out for every creep state on five images each in dendritic and interdendritic areas, after which the values were summarized. The mean value and the associated standard deviation are then calculated for each angle. This procedure is equivalent to the one described by Näth et al. [18]. The only difference is that the analysis here was not carried out on membranes, but on polished and etched samples.

The results of the line intersection method can be plotted polar so that the results are displayed as an ellipse. The vertical axis a refers to the γ-channel and γ′-ligament width. Since the γ-phase is removed for membrane production in the selective phase extraction, the γ-channel width is associated with the pore width in the nanoporous superalloy membrane. The horizontal axis c refers to the length of the γ- and γ′-ligaments, respectively. In the case of the line intersection method, it must be noted that due to a non-perfect alignment or the vertical connection of two γ-channels, an overestimation of the γ-channel width along the axis a may occur. In the case of the γ′-ligament length along the horizontal axis c, this can correspondingly lead to an underestimation.

## 3. Results

### 3.1. Creep Stress: 250 MPa

**Figure 2:**
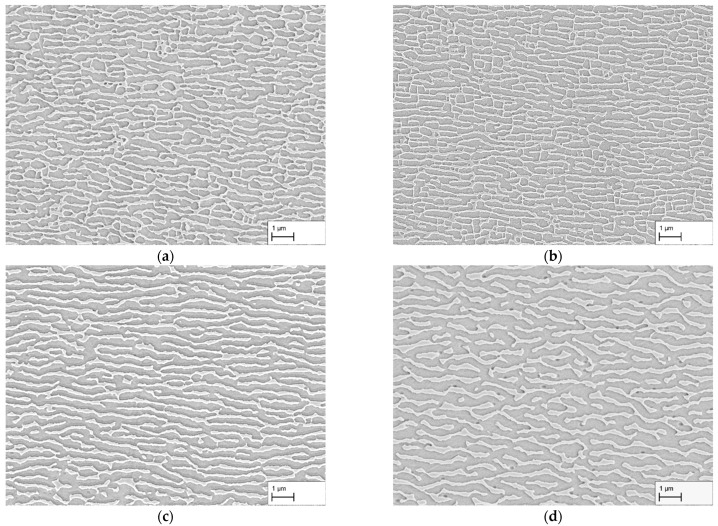
SEM images of dendritic areas after creep testing at 250 MPa/950 °C for durations of (**a**) 140 h, (**b**) 170 h, (**c**) 310 h, and (**d**) 506 h. Note, due to the etching of the γ′-phase, it appears darker than the γ-phase.

The results of the creep tests at a stress of 250 MPa are shown in Figure 2a–d. Exemplary images from the dendritic areas of the specimens are shown. After a creep time of 140 h, the microstructure in (a) shows an irregular raft structure. In many cases, vertical struts are present between the γ-channels. The individual channels are mostly not parallel to each other and have a curved shape. This contrasts with the microstructure in (b) after a creep period of 170 h. Compared to (a), the raft structure appears to be finer and the channels themselves are more regular and parallel in many places. Nevertheless, irregular areas and vertical connecting struts are also present here.

A further increase in the creep duration to 310 h results in a comparatively homogeneous raft structure with predominantly parallel γ-channels. Although the γ-channels have a slightly curved shape, they differ in appearance from the creep durations considered so far. There are also only a few vertical struts connecting the rafts. Overall, the structure appears very homogeneous and regular. After 506 h, the structure of the γ-channels changed in comparison to 310 h. The shape of the rafts is now more curved or bent and resembles a wavy shape. The structure is still homogeneous and regular, but the dimensions, especially the width of the channels, have increased.

**Figure 3:**
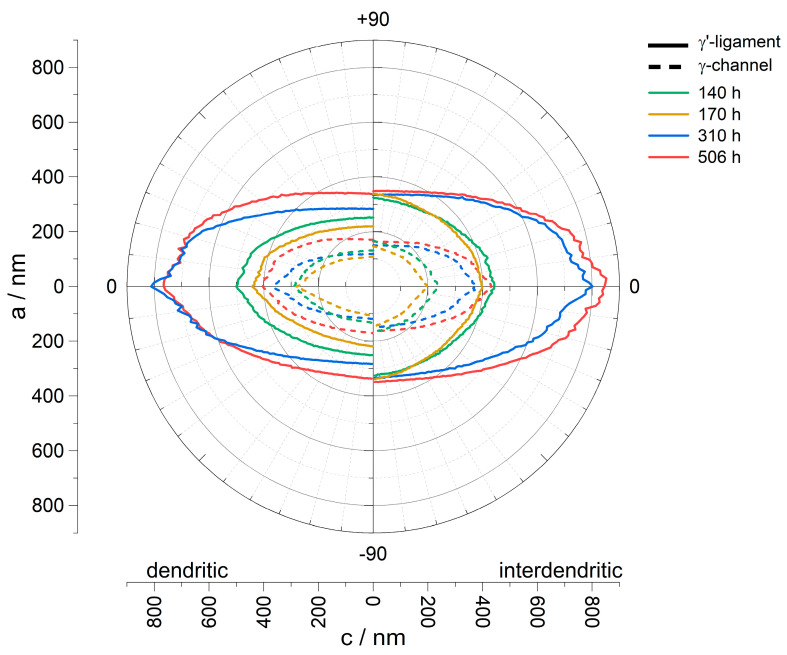
Structural ellipse of creep specimens A−CMSX−4/140h/250, A−CMSX−4/170h/250, A−CMSX-4/310h/250, and I−CMSX−4/506h/250. The values for γ-channels (dotted line) and γ′-ligaments (solid line) are shown. Furthermore, the left side shows the results of the dendritic, and the right side the results of the interdendritic areas. Each of these ellipses illustrates the associated length of the lines as a function of their orientation to the microstructure.

The development of the dimensions of the rafting structure can also be measured and plotted using the line intersection method. Figure 3 shows the structural ellipses of the creep tests performed at a stress of 250 MPa. The results have been plotted as polar plots. Accordingly, axis a refers to width, and axis c to length. The left half of the plot shows the results from dendritic regions, and the right half from interdendritic ones. The values are given in units of nanometers. Furthermore, the distinction between γ-channels and γ′-ligaments is made, where γ-channels are indicated as dashed lines and the solid lines refer to the γ′-ligaments. For further presentation of the results from the line intersection method, the values from Table 2 will be considered in more detail. These summarize the most important parameters for the characterization of the rafting structure. Values marked a refer to the vertical axis a and correspond to the results of the line intersection procedure in which the parallel lines were placed at an angle of 90° in the image. They are used to determine the width of the pores and ligaments. The label c corresponds to the angle 0°, i.e., along the axis c, and describes the length. The subscripts d and id indicate dendritic and interdendritic areas, respectively.

In both the dendritic and interdendritic regions, the progression of the γ-channel width is identical. The minimum in both cases is after a creep time of 170 h and is 108 ± 65 nm for γ-channels in the dendritic and 147 ± 123 nm in the interdendritic region. For the homogeneous structures in Figure 2c,d, 119 ± 43 nm and 171 ± 60 nm could be measured in the dendritic and 150 ± 66 nm and 164 ± 69 nm in the interdendritic regions. For the lowest creep duration of 140 h, γ-channel widths of 132 ± 70 nm in dendritic and 166 ± 137 nm in interdendritic regions are obtained. The γ-channel length shows a similar trend; the smallest channel length is found after 170 h and is 280 ± 273 nm in the dendritic and 198 ± 197 nm in the interdendritic region. It increases for the creep tests in the order 140 h, 310 h, and 506 h up to 403 ± 343 nm and 423 ± 354 nm for dendritic and interdendritic areas, respectively.

With regard to the width of the γ′-rafts, there are strong similarities to the γ-channels in the dendritic areas. The smallest width was also measured here after a creep time of 170 h and amounts to 219 ± 92 nm. This increases in the sequence 140 h, 310 h, and 506 h up to 337 ± 163 nm. In the interdendritic areas, the results are more constant. The minimum is 325 ± 163 nm after 140 h, after 170 h 338 ± 147 nm, after 310 h 334 ± 147 nm, and after 506 h 348 ± 170 nm.

Considering the interdendritic regions, a pronounced shape difference between the two ellipses for the shorter test durations (140 h, 170 h) and those for the longer ones (310 h, 506 h) is noticeable. While the ellipses show c/a-ratios of 2.50 and 2.58 for the γ-channels as well as 2.40 and 2.43 for the γ′-ligaments after 310 h and 506 h, respectively (see Table 2), the c/a-ratios after 140 h and 170 h are 1.43 and 1.35 for the γ-channels as well as 1.36 and 1.18 for the γ′-ligaments. This indicates that the interdendritic regions of the specimens with creep durations of 140 h and 170 h have a different microstructure compared to the dendritic regions after 140 h and 170 h as well as to the dendritic and interdendritic regions after 310 h and 506 h, respectively.

**Figure 4:**
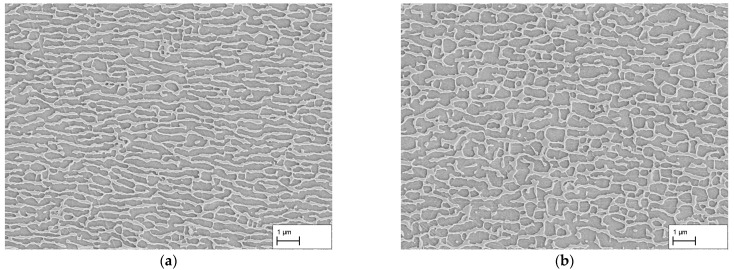

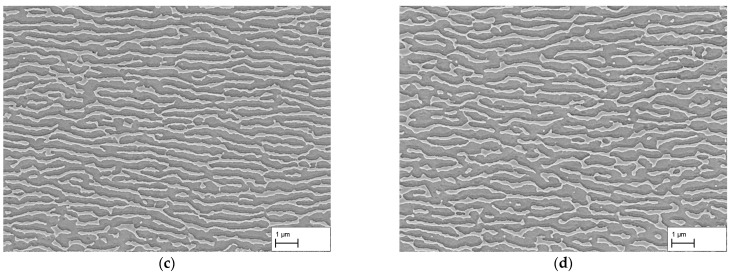
SEM images after creep testing at 250 MPa/950 °C for (**a**) dendritic and (**b**) interdendritic areas after 140 h; (**c**) dendritic and (**d**) interdendritic areas after 310 h. Note, due to the etching of the γ′-phase, it appears darker than the γ-phase.

In this context, Figure 4a–d show an example comparison of the dendritic and interdendritic regions after creep durations of 140 h and 310 h, respectively. In (a), the microstructure from a dendritic region can be seen. As described previously, the microstructure shows irregular γ′-rafts and γ-channels. In contrast, the microstructure imaged in (b) is from an interdendritic region and shows mostly isolated, coarsened γ′-cubes and elongated γ-channels. Overall, the structure shown is very inhomogeneous. If we now compare Figure 4c,d from dendritic and interdendritic regions after 310 h, we see that the microstructure in both regions has a directional appearance. Although the γ′-rafts and γ-channels in (c) are more uniform and finer than in (d), a fully developed raft structure is present in both figures. This comparison shows that dendritic and interdendritic regions display different stages of directional coarsening depending on the creep duration. This applies not only to the state after 140 h creep shown here, but also to the state after 170 h creep.

**Figure 5:**
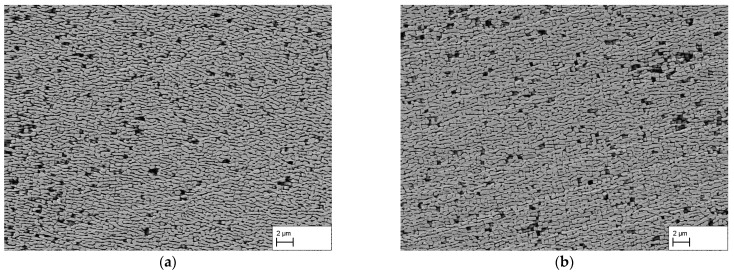

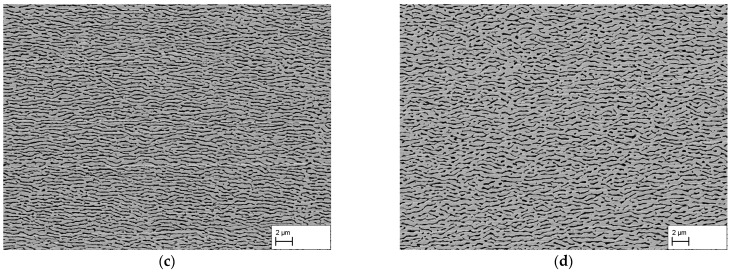
Membrane SEM images of dendritic areas after creep testing at 250 MPa/950 °C for durations of (**a**) 140 h, (**b**) 170 h, (**c**) 310 h, and (**d**) 506 h. Note, due to the dissolving of the γ-phase, pores appear black.

The obtained results for the dendritic and interdendritic areas after 140 h and 310 h creep duration show that, depending on the location, the raft formation process is at different stages. Since complete crosslinking is required for membrane production and a connected structure must be present for the subsequent application purpose, the surfaces of the membranes manufactured from the creep samples are examined in the following. Figure 5a–d show the membrane surfaces at dendritic areas after 140 h, 170 h, 310 h, and 506 h creep deformation. In both (a) and (b), holes or breakouts of the γ′-phase can be seen on the surface demonstrating that the γ′-phase was not completely connected at those locations. In contrast, in (c) and (d), the surfaces do not show any breakouts or irregularities. Thus, crosslinking of the γ′-phase is now complete everywhere. As previously observed in Figure 2c,d, a regular microstructure is present.

**Figure 6:**
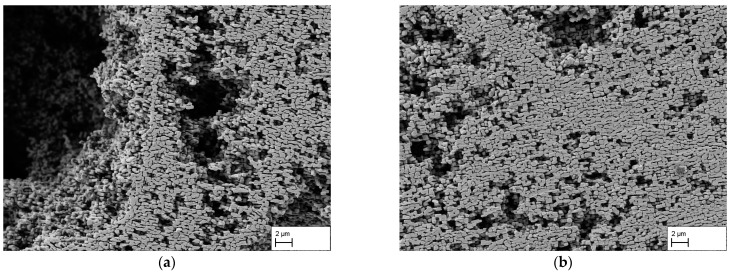

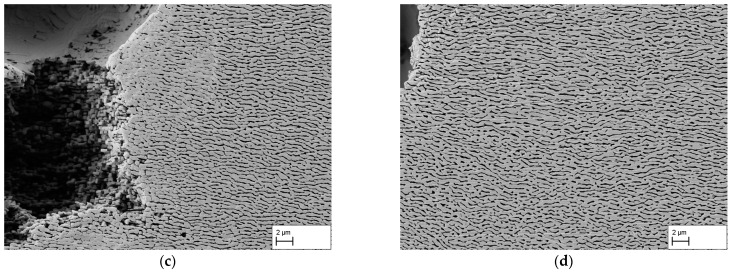
Membrane SEM images of interdendritic areas after creep testing at 250 MPa/950 °C for durations of (**a**) 140 h, (**b**) 170 h, (**c**) 310 h, and (**d**) 506 h. Note, due to the dissolving of the γ-phase, pores appear black.

Figure 6a–d show the membrane surfaces at interdendritic regions after 140 h, 170 h, 310 h, and 506 h. After a creep duration of 140 h, a fissured surface can be seen in (a). In several areas of the image, the γ′-phase is missing over a large area, and a kind of skeleton of the remaining γ′-phase forms a fissured structure. In addition, small breakouts can be seen in the areas of the remaining γ′-phase. In (b), a similar situation after 170 h can be seen. The large-scale breakouts of the γ′-phase are still present, but are smaller than in (a). Furthermore, in (b), there are also small breakouts of the γ′-phase between the ligaments. Overall, the structures on the surface of (a) and (b) look very similar. After a creep time of 310 h, the surface in (c) shows no breakouts of the γ′-phase except near larger casting pores. This can be attributed to the altered stress state around pores during creep deformation, hampering directional coarsening at certain locations. After 506 h of creep deformation, breakouts are absent everywhere (see Figure 6d).

**Figure 7:**
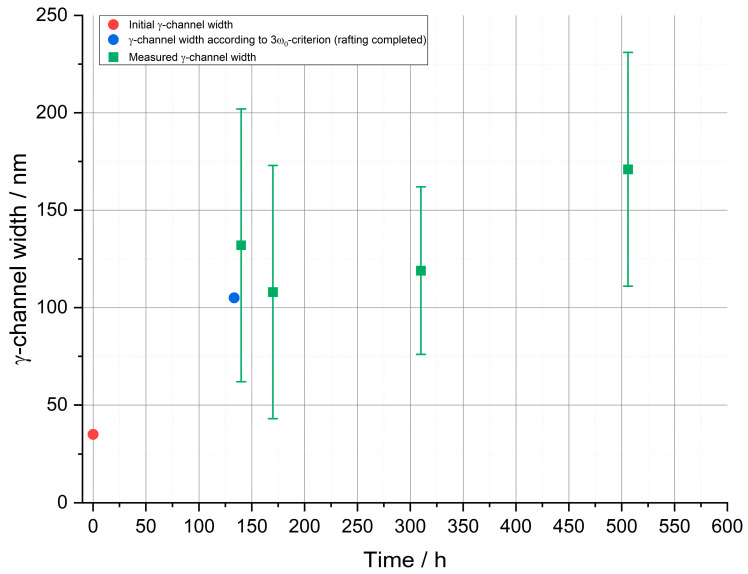
Summary of the γ-channel widths for the creep tests at a temperature of 950 °C and a stress of 250 MPa. The initial channel width in the precipitation heat-treated state, the minimum channel width after completion of the rafting process according to the $3w_{0}$-criterion and the measured channel widths (dendritic) of the creep tests are plotted.

Finally, it is possible to plot the results of this investigation for a stress of 250 MPa in a graph. Figure 7 shows the γ-channel width in nanometers as a function of the creep time in hours for the dendritic regions. Marked as a red circular dot is the initial channel width of 35 nm in the precipitation heat-treated condition. The blue point marks the result of the ${3w}_{0}$-criterion and represents the minimum channel width of 105 nm when raft formation is just completed. According to Epishin et al. [19], this occurs in the dendritic regions after 133 h for the stress of 250 MPa at 950 °C. In green, the γ-channel widths measured in the dendritic regions are indicated with the corresponding standard deviations. The diagram visualizes the results from Table 2. The obtained γ-channel width for the creep time of 140 h is 132 ± 70 nm, which is higher than the value of 105 nm given by the $3w_{0}$-criterion. After 170 h, the value for the γ-channel width decreases to 108 ± 65 nm and is thus very close to the value of the ${3w}_{0}$-criterion. For the creep duration of 310 h, the value increases slightly to 119 ± 43 nm and for 506 h to 171 ± 60 nm. Furthermore, it becomes clear that the γ-channel width in general increases with increasing creep duration. Nevertheless, the results for the creep duration of 140 h show a larger γ-channel width than after 170 h and 310 h. On the one hand, this can be attributed to the measurement uncertainty (see large standard deviations); on the other hand, the cross-links in the vertical direction and the non-parallel alignment of the γ-channels and γ′-ligaments after 140 h have an influence on the measurements. This can lead to an overall overestimation of the γ-channel width. With increasing creep time, the γ-channel width increases, the cross-links decrease and the microstructure aligns better. If the decrease in the cross-links and the alignment of the γ′-ligaments and γ-channels outweigh the real channel widening, the used measurement method may result in a minimum as observed after 170 h creep duration.

In summary, the results of the investigation at a stress of 250 MPa show that dendritic and interdendritic regions coarsen at different rates. With the measurement method used in this study, the γ-channel width of 105 nm predicted via the $3w_{0}$-criterion could not be achieved in dendritic regions after a creep period of 140 h. Firstly, the still existing crosslinking between the γ-channels can be stated as a reason here, and secondly, the non-parallel alignment influences the results of the line intersection method. On the basis of the measurements and taking into account the influencing factors, however, it can be concluded that the actual γ-channel width is overestimated and that the real channel width must be smaller than the measured 132 ± 80 nm. Nevertheless, the results show that an approximation to the γ-channel width established by the $3w_{0}$-criterion is possible via variation in creep duration. Using two-dimensional SEM images, the smallest γ-channel width with a complete directionally coarsened microstructure in dendritic and interdendritic regions could be observed after 310 h, whereas the creep durations of 140 h and 170 h show directionally coarsened structures only in the dendrites and after 506 h larger structures have already emerged. Considering the later application in premix membrane emulsification, membranes were prepared from the tested creep samples. The results from the observation of the surfaces show that the membranes from the creep samples with a creep time of 140 h and 170 h do not have complete crosslinking in dendritic and interdendritic areas. While in dendritic areas, isolated parts of the γ′-phase fell out during the dissolution of the γ-phase, the interdendritic areas of both membranes show more extensive breakouts of the γ′-phase. Complete crosslinking can be observed in dendritic areas after a creep time of 310 h. Contrary to the results from the longitudinal sections, breakouts can also be seen in interdendritic areas in the region around the casting pores. Note that this does not hinder membrane application so that a creep duration of 310 h is considered optimal when the finest possible pores are the objective. Finally, after a creep time of 506 h, dendritic and interdendritic areas show complete crosslinking everywhere.

### 3.2. Creep Stress: 183 MPa and 140 MPa

**Figure 8:**
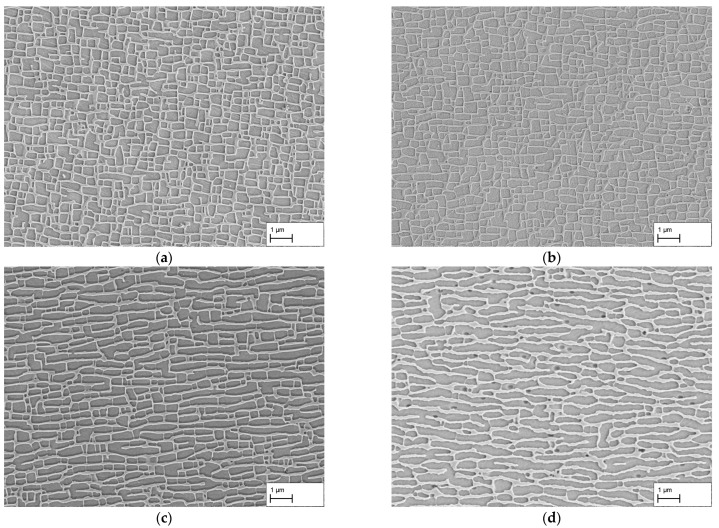
SEM images of dendritic areas after creep testing at 183 MPa/950 °C for durations of (**a**) 140 h, (**b**) 170 h, (**c**) 310 h, and (**d**) 506 h. Note, due to the etching of the γ′-phase, it appears darker than the γ-phase.

Figure 8a–d show the dendritic microstructures at a stress of 183 MPa as a function of time. In (a), after 140 h, as well as in (b), after 170 h, a cubic precipitation structure can be seen. In contrast to (a), the structure in (b) appears more inhomogeneous, and the γ′-precipitates are in many places no longer cube-shaped but rectangular. In (c), a raft structure is already present after 310 h, and a large number of vertical γ-channels continues to be present, connecting the horizontal and parallel γ-channels. The structure in (c) is comparatively homogeneous. Measurements after 310 h result in a γ-channel width of 141 ± 130 nm. In (d), the dimensions of the γ-channels have increased after 506 h. Moreover, the γ′-rafts have increased in size. Vertical γ-channels are still present but they are more irregular with respect to their shape or run increasingly in a curved form. Measurements after 506 h result in a γ-channel width of 147 ± 85 nm.

**Figure 9:**
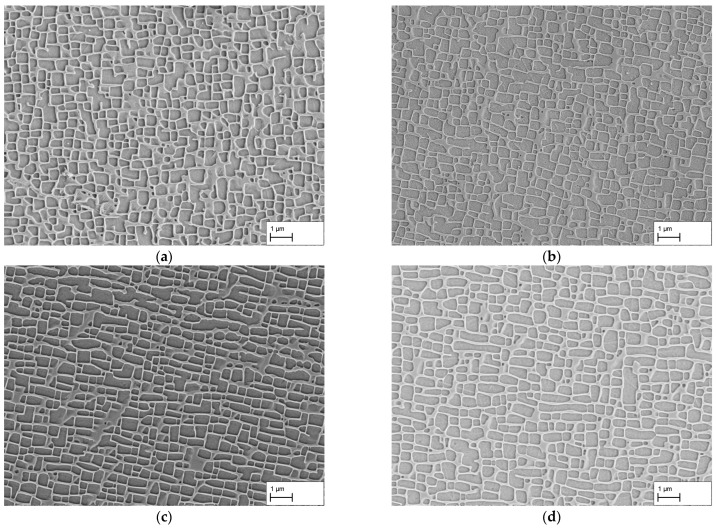
SEM images of dendritic areas after creep testing at 140 MPa/950 °C for durations of (**a**) 140 h, (**b**) 170 h, (**c**) 310 h, and (**d**) 506 h. Note, due to the etching of the γ′-phase, it appears darker than the γ-phase.

Finally, Figure 9a–d show the microstructures of the creep specimens with a stress of 140 MPa. In (a), after 140 h, and (b), after 170 h, a cubic γ′-precipitate structure can be seen; although, the γ′-particles are less regularly arranged in (b) than in (a), so that the microstructure appears more inhomogeneous. In (c), after a creep time of 310 h, the first rectangular γ′- particles are visible in addition to cubic γ′-precipitates. In comparison to (d), it is noticeable that the γ′-particles are very angular, whereas in (d), they have lost their angularity after 510 h. At the same time, several elongated γ′-precipitates are visible. Nevertheless, it is not possible to speak of a raft structure here.

## 4. Discussion

By loading the creep specimens along the [001]-orientation at constant temperature and stress, a directionally coarsened γ/γ′-microstructure was observed after a sufficiently long creep period. This is an N-type raft structure that occurs at negative misfit δ of the alloy. The alloy CMSX-4 has a negative misfit δ at room temperature, which becomes more negative with increasing temperature. The formation of type-N rafts is thus consistent with results from the literature, for example [6,7,8,14,19,20,26,27,28,29]. For a stress of 250 MPa, a directionally coarsened microstructure in the dendritic and interdendritic regions was observed at a creep time of 310 h. Moreover, after 140 h and 170 h, rafting of the γ′-precipitates is essentially complete in the dendritic domains. However, the interdendritic areas show only partial areas in which directional coarsening, i.e., raft formation, has taken place (see Figure 4). According to Reed et al. [14] the reason for these different rates of raft formation is the chemical imbalance between dendritic and interdendritic areas, which leads to different γ′-volume contents, different misfits, and chemical compositions of the phases. Aluminum and tantalum, in particular, preferentially accumulate in interdendritic regions, so that the γ′-volume content is greater here than in dendritic regions. This leads to the γ-channels being narrower, which results in greater creep resistance. Additionally, Völkl et al. have shown that the misfit is more positive in the interdendritic regions than in the dendritic ones in the case of CMSX-4 [30]. These aspects explain the observation made here that raft formation in the interdendritic regions is delayed compared to the dendritic ones, which is why the creep durations of 140 h and 170 h are not suitable to obtain a completely directionally coarsened microstructure. Nevertheless, the results show that approximating the optimal creep duration for a directionally coarsened microstructure via the channel widening rate ${\dot{w}}_{1}$ determined by Epishin et al. [20] is reasonable, since the results can be used as a starting point for further experiments. Since the channel widening rate was determined in dendritic regions and the results of our investigation show raft structures in the same already after 140 h, a consistency is given. However, because the raft structure after 140 h and 170 h contains vertical γ-channels to a significant extent, there is a considerable overestimation of the γ-channel width using the line intersection method, especially after 140 h, which results in a γ-channel width of 132 ± 70 nm. Taking this into account, the actual channel width appears to be close to three times its initial value $3w_{0}$= 35 nm, in agreement with the $3w_{0}$-criterion for just-completed raft formation proposed by Epishin et al. [19]. Note that the same holds true for the width of 108 ± 65 nm measured after 170 h.

The results from Figure 7 show that with the completion of raft formation in the dendritic regions at about 140 h to 170 h, the widening rate of the γ-channels decreases considerably. This is also consistent with the results of Epishin et al. [20] and has been explained by the higher stability of the plate-like γ′-structure compared to the cube-like one as well as different coarsening mechanisms associated with these different γ′-morphologies. Considering the fabrication of nanoporous superalloy membranes where a bicontinuous γ/γ′-microstructure is required prior to selective phase extraction, the slowed coarsening kinetics is of great benefit. It allows creep durations to be selected where rafting is completed not only in the dendritic but also in the interdendritic regions with limited further coarsening of the already-rafted dendritic areas. In this respect, a creep duration of 310 h at 250 MPa appears to be favorable. Then, the interdendritic regions are fully rafted (see Figure 4d) while the γ-channel width in the dendritic ones is still close to the minimum possible value (see Figure 7). Even though isolated breakouts still occur at casting pores in the interdendritic regions (see Figure 6c), they are not expected to affect the mechanical strength or functionality of the membrane in any appreciable way because they neither significantly reduce the membrane’s cross-section nor do they cause a short-circuit for the flow of a medium through the membrane.

The other results for the stresses of 183 MPa and 140 MPa show that the raft formation process becomes less complete with decreasing stress at constant creep duration (see Figure 8 and Figure 9). According to Kamaraj et al. [6] and Pollock et al. [7], the driving force for the raft formation process is the increase in dislocation density in the γ-channels when the creep specimen is loaded. The introduced dislocations reduce or increase the misfit δ in the horizontal and vertical channels, respectively, and thus the associated elastically stored energy. Due to the resulting change in thermodynamic driving forces and chemical potentials, raft formation occurs, eliminating the energetically less favorable vertical γ′-channels. Thus, the greater the stress in the creep test, the more dislocations are introduced into the γ-channels within a certain time period and the greater the thermodynamic driving force for the raft formation process. This is the reason for the increasingly incomplete raft formation process with decreasing stress at constant temperature and creep duration. It can also be inferred from the results that the channel widening rate during the process of raft formation becomes smaller with decreasing stress, which is in agreement with the results of Epishin et al. [20]. This is simply due to the fact that the dislocations in the γ′-channels, and with them the thermodynamic driving force for rafting, evolve more slowly with decreasing stress.

Another observation was made during the creep test at 183 MPa and 506 h. According to Epishin’s $3w_{0}$-criterion and the stress-dependent widening rate [20], the calculated minimum channel width of 105 nm would have to be reached after a longer creep time at 183 MPa than at a50 MPa. In certain respects, this is consistent with the experimental results. They show that raft formation is complete after 506 h/183 MPa in the dendritic areas in a similar way as after 140 h and 170 h at 250 MPa (compare Figure 8d with Figure 2a,b) with numerous vertical γ-channels still present in all these cases. However, as the width of the horizontal channels is already 141 ± 130 nm in the former case, i.e., larger than $3w_{0}$ and the widths after 140–170 h/250 MPa, there is an additional factor influencing raft formation at this point. It is reasonable to assume that, as already observed by Epishin et al. [20], undirectional coarsening of the γ/γ′-microstructure by Ostwald ripening takes place in parallel to the ongoing raft formation process, becoming the more important the slower the rafting is, i.e., the lower the applied stress. For this reason, the minimum possible raft width increases with decreasing stress and the $3w_{0}$-criterion has to be seen as a lower limit in the absence of undirectional coarsening. This is also suggested by the observations of Chen et al. [31] who reasoned that the increase in thickness of the γ′-rafts is related to Ostwald ripening as a function of the initial size of the γ′-particles and the coarsening rate. This results not only in the coarsening of the γ′-rafts but also of the γ-channels. It can be concluded that the channel widening rate at 183 MPa is so low that undirectional coarsening already takes place to a significant extent during the raft formation process. Considering the application of interest here, it means that a creep stress of 250 MPa is favored over the lower ones inspected here. As the achieved channel width is already close the lower limit of $3w_{n}$, even higher stresses are expected to be of limited benefit.

## 5. Conclusions

In this study, the γ-channel width was investigated as a function of creep duration and stress using staged creep specimens. Stresses of 250 MPa, 183 MPa, and 140 MPa and creep durations of 140 h, 170 h, 310 h, and 506 h were considered. The test temperature was 950 °C in all cases. The following conclusions can be drawn from this investigation:

1. The use of stepped creep specimens with three stress ranges allows the simultaneous investigation of different creep states within one creep test. This can reduce material usage and make it easier to find the optimum combination of temperature, stress, and creep duration.
2. The $3w_{0}$-criterion is suitable for approximating the minimum γ-channel width in the dendritic regions of the single-crystalline Ni-based superalloy CMSX-4 achieved at the point in time when rafting is just completed. The calculated minimum γ-channel width of 105 nm could be essentially reached after 170 h/250 MPa with a measured γ-channel width of 108 ± 65 nm in the dendritic regions. Due to the chemical imbalances between dendritic and interdendritic regions, no raft structure was present in the interdendritic regions at this point in time, which does not allow for the use of this microstructure for membrane fabrication.
3. The combination of a temperature of 950 °C, a stress of 250 MPa, and a creep time of 310 h achieves the requirements we set for a membrane structure, leading to a γ-channel width of 119 ± 43 nm in dendritic and 150 ± 66 in interdendritic regions and complete crosslinking. Further characterizations such as mechanical strength or emulsification behavior of nanoporous superalloy membranes produced by these parameters have to be performed.
4. In combination with the test temperature of 950 °C, the stresses 183 MPa and 140 MPa do not lead to suitable microstructures for the fabrication of membranes for premix membrane emulsification as the lower rafting speed in combination with undirectional coarsening results in larger γ-channels than required.
5. The use of previous research results to investigate the microstructure development of CMSX-4 in the high-temperature range could be successfully transferred to the field of membrane development and contribute to further optimization of Ni-based nanoporous membranes. For future application in the field of premix membrane emulsification and the production of colloidal lipid-based excipients, further experiments have to be carried out, for example, on the mechanical properties or resulting droplet size.

## Figures and Tables

**Table 1:** Loading conditions and geometrical details of the stepped creep test samples; θ is the misorientation angle between the crystallographic [001]-orientation and the long axis of the single crystal rods.

Specimen ID	Cross Section (mm^2^)	θ (°)	Stress (MPa)	Duration (h)
A-CMSX-4/140h/250	28.27	9.9	250	140
A-CMSX-4/170h/250				
A-CMSX-4/310h/250		9.8		170
I-CMSX-4/506h/250		9.6		310
		6–10		506
A-CMSX-4/140h/183	38.48	9.9	183	140
A-CMSX-4/170h/183		9.8		170
A-CMSX-4/310h/183		9.6		310
I-CMSX-4/506h/183		6–10		506
A-CMSX-4/140h/140	50.26	9.9	140	140
A-CMSX-4/170h/140		9.8		170
A-CMSX-4/310h/140		9.6		310
I-CMSX-4/506h/140		6–10		506

**Table 2:** Results of the line intersection measurements of the longitudinal sections of the creep tests with a stress of 250 MPa at a test temperature of 950 °C. The values for the vertical axis a and horizontal axis c of the structural ellipse are given. The additions d and id refer to the analyzed dendritic and interdendritic areas; the additions γ- and γ′-channels po denote the respective structure.

	A-CMSX-4/140h/250	A-CMSX-4/170h/250	A-CMSX-4/310h/250	I-CMSX-4/506h/250
c_d, γ-channel_	290 (261)	280 (273)	363 (329)	403 (343)
c_id, γ-channel_	238 (232)	198 (197)	375 (326)	423 (545)
a_d, γ-channel_	132 (70)	108 (65)	119 (43)	171 (60)
a_id, γ-channel_	166 (137)	147 (123)	150 (66)	164 (59)
c_d, γ′-channel_	500 (391)	443 (347)	812 (786)	766 (582)
c_id, γ′-channel_	442 (334)	398 (236)	801 (679)	845 (691)
a_d, γ′-channel_	251 (106)	219 (92)	284 (101)	337 (163)
a_id, γ′-channel_	325 (163)	338 (169)	334 (147)	348 (170)
(c/a)_d, γ-channel_	2.20	2.59	3.05	2.36
(c/a)_id, γ-channel_	1.43	1.35	2.50	2.58
(c/a)_d, γ′-ligament_	1.99	2.02	2.86	2.27
(c/a)_id, γ′-ligament_	1.36	1.18	2.40	2.43

## Data Availability

Not applicable.
